# The Role of *ccpA* in Nitrogen Source-Induced Heat and Oxidative Stress Tolerance Changes in *Lacticaseibacillus rhamnosus*

**DOI:** 10.3390/foods14223894

**Published:** 2025-11-14

**Authors:** Mengting Li, Haohao Cheng, Qiming Li, Yue Sun, You Wu, Haikang Wang, Yunchao Wa, Dawei Chen, Chengran Guan, Yujun Huang, Ruixia Gu, Chenchen Zhang

**Affiliations:** 1 School of Food Science and Engineering, Yangzhou University, Yangzhou 225127, China; 18205256509@163.com (M.L.); sdwfchh@163.com (H.C.); 13773444357@163.com (Y.S.); 13961079026@163.com (Y.W.); 18663062545@163.com (H.W.); 18252773850@163.com (Y.W.); dwchen@yzu.edu.cn (D.C.); crguan@yzu.edu.cn (C.G.); yjhuang@yzu.edu.cn (Y.H.); guruixia1963@163.com (R.G.); 2 Jiangsu Provincial Key Laboratory of Probiotics and Dairy Deep Processing, Yangzhou University, Yangzhou 225127, China; 3 Department of Medical Technology, Guangxi Health Science College, Nanning 530021, China; 4 Dairy Nutrition and Function Key Laboratory of Sichuan Province, New Hope Dairy Co., Ltd., Chengdu 610023, China; liqm@newhope.cn

**Keywords:** lactic acid bacteria, carbon catabolite repression, tryptone, stress tolerance, co-regulation

## Abstract

The viable bacterial count is a crucial quality indicator for lactic acid bacteria (LAB) starters and fermented foods. Metabolic activity is an integral component of stress tolerance pathways. *Lacticaseibacillus rhamnosus* exhibits enhanced heat and oxidative stress tolerance in tryptone-free media. To investigate the stress tolerance mechanisms from a metabolic perspective, the heat and oxidative stress tolerance and transcriptomic changes in *L. rhamnosus* hsryfm 1301 and its *ccpA* deficient strain (ΔccpA) were analyzed under different nitrogen source conditions. Slower growth, decreased heat stress tolerance, and enhanced oxidative stress tolerance were observed in ΔccpA in MRS. Compared to the wild-type strain, 260 genes were upregulated and 55 genes were downregulated in ΔccpA, mainly including carbon source transport and metabolism genes, but no typical stress tolerance genes. The regulation of *pfk*, *pyk*, *dnaK*, and *groEL* was different from that in other lactic acid bacteria. The pathways related to acetate production were regulated solely by *ccpA* deletion, while *dnaK*, *groEL*, and de novo pyrimidine synthesis genes were only regulated by tryptone. Fatty acid and purine synthesis genes and *glmS* were co-regulated by *ccpA* and tryptone. The deletion of *ccpA* eliminated the nitrogen source-induced oxidative stress tolerance changes. It was found that *ccpA* in *L. rhamnosus* can affect both carbon and nitrogen source metabolism, altering stress tolerance.

## 1. Introduction

Lactic acid bacteria (LAB) are widely used in food production. Their metabolic activities impart unique flavors, textures, and extended shelf life to food, making it more acceptable and digestible for humans [1]. Moreover, the live bacterial cells, upon entering the human body, can exert probiotic effects such as regulating gut microbiota, modulating immunity, and producing beneficial substances [2]. Therefore, the viable bacterial count is a crucial quality indicator for LAB starters and fermented foods.

LAB have a long history of application in food fermentation. With the confirmation of their probiotic functions, their forms in food have become increasingly diverse (including starters, fermentation broths, bacterial powders, cell pastes, granules, etc.). During production, transportation, and storage, LAB encounter more complex stress scenarios (such as spray drying) involving acid, oxidation, heat, osmotic pressure, and freezing, posing greater challenges to maintaining their viability [3]. Therefore, stress tolerance mechanisms of LAB have consistently been a research hotspot in this field.

Stress tolerance in microbes is a complex environmental response. Using random gene inactivation, comparative genomics, transcriptomics, and proteomics, researchers have identified numerous typical genes and pathways like *ctsR*, *hrcA*, *lexA*, *relA*, and two -/multi-component systems [4,5,6,7]. In recent years, omics data have revealed that among the hundreds of genes responding to a single stressor, a significant proportion are often metabolism-related genes. Moreover, the regulation of these genes is more sensitive and widespread compared to typical stress tolerance genes [8,9]. This indicates that metabolic activities not only play a crucial role in microbial growth but also an integral component of stress tolerance pathways.

*Lacticaseibacillus rhamnosus* is widely distributed in the human gut and breast milk [10,11], recognized as one of the most extensively studied and applied probiotics. Strains such as hsryfm 1301, GG, HN001, LYO, and SP1 exhibit enhanced heat and oxidative stress tolerance under low-tryptone conditions. Moreover, amino acids including glutamate, methionine, alanine, histidine, isoleucine, and phenylalanine significantly reduce their heat and oxidative stress tolerance [12]. This indicates that nitrogen metabolism critically modulates the stress tolerance of *L. rhamnosus*. However, the nitrogen metabolism regulator GlnR only affects induced heat and oxidative stress tolerance (e.g., via sublethal preadaptation) and has no impact on direct heat/oxidative shock resistance [13].

CcpA, the global regulator of carbon catabolism, is another extensively studied metabolic regulatory element in LAB. In the presence of glucose, CcpA binds with cofactors such as fructose-1,6-bisphosphate and phosphate groups to repress the utilization of non-preferred carbon sources [14]. Additionally, CcpA performs other regulatory functions. In lactic acid bacteria such as *Lactiplantibacillus plantarum*, *Lactobacillus delbrueckii*, and *Lactococcus lactis*, inactivating the *ccpA* gene affects the expression of over a hundred genes. These genes are involved in carbon and nitrogen source utilization, stress tolerance, and metabolic flux redistribution [15,16,17,18]. Significantly, both oxidative stress and heat stress tolerance in these LAB is controlled by CcpA [19].

This study focused on *L. rhamnosus* hsryfm 1301 and its *ccpA* deficient strain. By analyzing their stress tolerance capabilities under different nitrogen source conditions combined with transcriptome data, we aimed to investigate whether the *ccpA* gene plays a role in nitrogen source-induced alterations of heat stress and oxidative stress tolerance in *L. rhamnosus*.

## 2. Materials and Methods

### 2.1. Bacterial Strains, Plasmids, and Growth Conditions

The strains used in this study are listed in Table 1. *L. rhamnosus* hsryfm 1301 was firstly isolated from the gut of a centenarian, possessing valuable probiotic properties [20,21]. *L. rhamnosus* strains were cultured in de Man, Rogosa and Sharpe (MRS) broth (2% (*v*/*v*) inoculation) at 37 °C under static incubation. Plasmid construction was implemented using *Escherichia coli* XL1-Blue as the host [13].

The base formulation for MRS medium (tryptone 10 g L^−1^) was as described in a previous study [12]. NP-MRS (tryptone-free MRS) were prepared according to our previous studies [22].

**Table 1:** The strains and plasmids used in this study.

**Strains**	**Genotype or Characteristics**
*L. rhamnosus* hsryfm1301	Chinese centenarian; CGMCC No. 8545
*L. rhamnosus* ΔccpA	*ccpA* mutant of *L. rhamnosus* hsryfm 1301
*L. rhamnosus* ∆ccpA/pccpA(L.r.)+	*L. rhamnosus* ΔccpA with pccpA(L.r.)+
*L. rhamnosus* ∆ccpA/pccpA(L.p.)+	*L. rhamnosus* ΔccpA with pccpA(L.p.)+
*L. paracasei* PC-01	Commercially available drink Youyi C
*E. coli* XL1-Blue	SHBCC, Shanghai, China
**Plasmids**	**Genotype or Characteristics**
pUC19e	Erm^r^, pUC19 derivative [12]
pUC19e-ccpAUD	Erm^r^, pUC19e derivative with upstream and downstream sequences of *ccpA* gene
pMG36e	Erm^r^ [23]
pccpA(L.r.)+	Erm^r^, pMG36e derivative with *ccpA* gene of *L. rhamnosus* hsryfm 1301
pccpA(L.p.)+	Erm^r^, pMG36e derivative with *ccpA* gene of *L. paracasei* PC-01

### 2.2. Construction of Plasmids

The plasmids used in this study are listed in Table 1. Primers used are listed in Table 2. PrimeSTAR Max DNA polymerase, restriction enzymes, T4 DNA ligase (TaKaRa, Beijing, China), Taq polymerase, and ClonExpress Ultra One Step Cloning Kit (Vazyme, Nanjing, China) were used according to standard procedures.

The suicide vector, pUC19e-ccpAUD was constructed as previously described [13]. The upstream (amplified with primers Eco-ccpaupF and ccpaupR) and downstream (amplified with primers ccpadownF and hind-ccpadownR) sequences of the ccpA gene of *L. rhamnosus* hsryfm 1301 were PCR spliced and inserted into pUC19e.

The pMG36e fragment was amplified with primers pMG36eF and pMG36eR. The *ccpA* gene of *L. rhamnosus* hsryfm 1301 (1301-ccpA, including the promoter) was amplified with primers Eco-1301ccpAF and Hind-1301ccpAR. The pMG36e and 1301-ccpA fragments were digested with *Eco*R I and *Hind* III and ligated with T4 DNA ligase, resulting in pccpA(L.r.)+. The *ccpA* gene (FGL-ccpA, including the promoter) of *Lacticaseibacillus paracasei* PC-01 was amplified with primers 36e-FGL-ccpAF and 36e-FGL-ccpAR, and pccpA(L.r.)+ was constructed by fusing the pMG36e and FGL-ccpA fragments with the ClonExpress Ultra One Step Cloning Kit.

### 2.3. Gene Deletion and Complementation

The marker-free deletion of *ccpA* gene in *L. rhamnosus* hsryfm 1301 was implemented by using pUC19e-ccpAUD. The single-crossover mutants were identified using PCR with the primer pairs ccpaTestF/RV-M and ccpaTestR/M13c-F, and the *ccpA* mutants with double-crossover were identified using PCR with the primer pair ccpaTestF/ccpaTestR.

To complement the *ccpA* gene, pccpA(L.r.)+ and pccpA(L.p.)+ were, respectively, transformed into the *ccpA* mutant of *L. rhamnosus* hsryfm 1301 by electroporation (2500 V, 25 µF, 400 Ω).

### 2.4. Growth Investigation of L. rhamnosus Strains

Before the growth investigation, *L. rhamnosus* strains were activated by overnight incubation (20 h). The cultures were diluted 50-fold in 5 mL of fresh MRS broth, and growth curves were measured using a microbiological growth analyzer (Bioscreen C, LabSystems, Helsinki, Finland).

### 2.5. Detection of Heat Stress and Oxidative Stress Tolerance

After overnight incubation, *L. rhamnosus* hsryfm 1301, ΔccpA, ∆ccpA/pccpA(L.r.)+, and ∆ccpA/pccpA(L.p.)+ were diluted 50-fold in 5 mL of fresh MRS and NP-MRS broth, and incubated at 37 °C. OD_600_ values were measured using a biophotometer (BioPhotometer Plus, Eppendorf, Germany). Tolerance detection was performed when the OD_600_ reached 1.8–2.0 (in exponential phase). The treatment conditions of heat stress were 53 °C 1 h, 54 °C 1 h, 55 °C 1 h. The treatment conditions of oxidative stress were 2.0 mmol L^−1^ H_2_O_2_ 1 h, 3.0 mmol L^−1^ H_2_O_2_ 1 h, and 4.0 mmol L^−1^ H_2_O_2_ 1 h. The viable counts of the samples before and after the treatment were measured as previously described, and the survival rates were calculated [12,24].

### 2.6. RNA Isolation, and RNA Sequencing (RNA-seq)

After overnight incubation, *L. rhamnosus* hsryfm 1301 was diluted 50-fold in 50 mL of fresh MRS (YM group) and NP-MRS (YN group), respectively, and so was *L. rhamnosus* ΔccpA (QM and QN group). When the OD_600_ values reached 1.8–2.0, RNA isolation, library construction and RNA-seq of the samples were performed according to a previous study [22].

### 2.7. Mapping Reads to the Reference Genome and Normalized Gene Expression

Mapping reads to the reference genome were performed according to a previous study [22,25].

### 2.8. Differential Expression Analysis and Kyoto Encyclopedia of Genes and Genomes (KEGG) Enrichment Analysis

Differential expression and KEGG enrichment analyses were performed as previously described [22]. Transcripts with |log_2_FoldChange| > 1 and *p* value < 0.05 were considered as differentially expressed genes (DEGs). Pathways with a *p* value ≤ 0.05 were considered significantly enriched [26].

### 2.9. Nucleotide Sequence Accession Numbers

The raw sequence data have been deposited in the Genome Sequence Archive at National Genomics Data Center [27,28], (GSA: CRA027319, CRX1820520-CRX1820531; publicly accessible at https://ngdc.cncb.ac.cn/gsa, accessed on 9 October 2025).

### 2.10. Statistical Analysis

Growth investigation, survival rate measurement, and RNA sequencing were repeated three times. Survival rates and fragments per kilobase of exon model per million mapped fragments (FPKM) were analyzed with GraphPad Prism (Version 9.0.0, GraphPad Software, San Diego, CA, USA) using one-way ANOVA with Tukey’s post hoc multiple comparison test (*p* < 0.05).

## 3. Results

### 3.1. Deletion and Complementation of ccpA Gene in L. rhamnosus Hsryfm 1301

**Figure 1:**
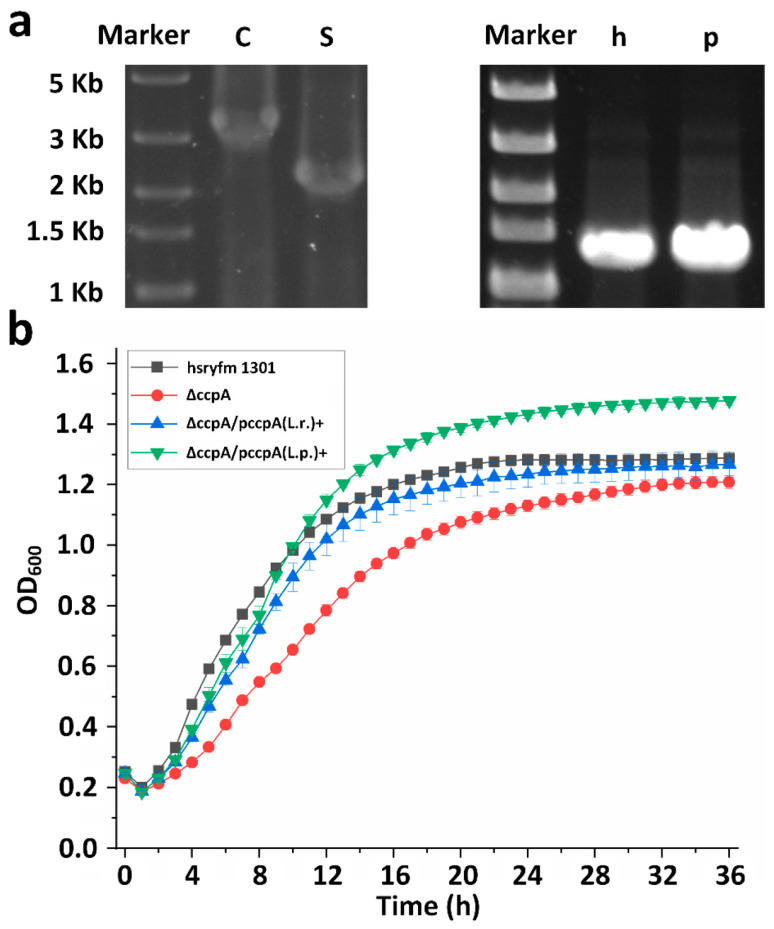
Deletion and complementation of the *ccpA* gene. (**a**) Left: Verification of the knockout of the *ccpA* gene with primers ccpaTestF and ccpaTestR. C, *L. rhamnosus* hsryfm 1301; S, strain with double-crossover. Right: Verification of the complementation of the *ccpA* gene from *L. rhamnosus* hsryfm 1301 (h) or *L. paracasei* PC-01 (p). (**b**) Growth curves of *L. rhamnosus* hsryfm 1301, ΔccpA, ∆ccpA/pccpA(L.r.)+ and ∆ccpA/pccpA(L.p.)+.

An in-frame marker-free deletion of *ccpA* gene was implemented in *L. rhamnosus* hsryfm 1301, generating *L. rhamnosus* ΔccpA. Using the primer pair ccpaTestF/ccpaTestR, a 3292 bp band should be amplified from *L. rhamnosus* hsryfm 1301. Given *ccpA*’s coding sequence is 1002 bp, this primer pair should amplify a 2290 bp band from its *ccpA* deficient strain. The electrophoresis of *L. rhamnosus* hsryfm 1301 and the mutant matched expectations (Figure 1a), confirming the successful knockout of the *ccpA* gene. This *ccpA* deficient strain was designated as *L. rhamnosus* ΔccpA. With reference to the first derivative of OD_600_, *L. rhamnosus* hsryfm 1301 entered the stationary phase at 24 h, while ΔccpA exhibited a delayed entry into the stationary phase at 35 h. (Figure 1b). Although the growth rate of ΔccpA was slower, its final growth amount was not significantly affected. The *ccpA* sequences including their promoters from *L. rhamnosus* and *L. paracasei* are 1243 bp and 1250 bp in length, respectively. PCR results confirmed that both sequences were successfully complemented into ΔccpA, constructing *L. rhamnosus* ∆ccpA/pccpA(L.r.)+ and *L. rhamnosus* ∆ccpA/pccpA(L.p.)+ (Figure 1a). Growth curve analysis indicated that the growth rates of both complemented strains were restored to the level of the wild-type strain (Figure 1b). *L. rhamnosus* ∆ccpA/pccpA(L.p.)+ exhibiting higher growth, which might result from the difference between the genes from *L. rhamnosus* and *L. paracasei*.

### 3.2. Impact of ccpA Gene Knockout on Heat and Oxidative Stress Tolerance in L. rhamnosus

**Figure 2:**
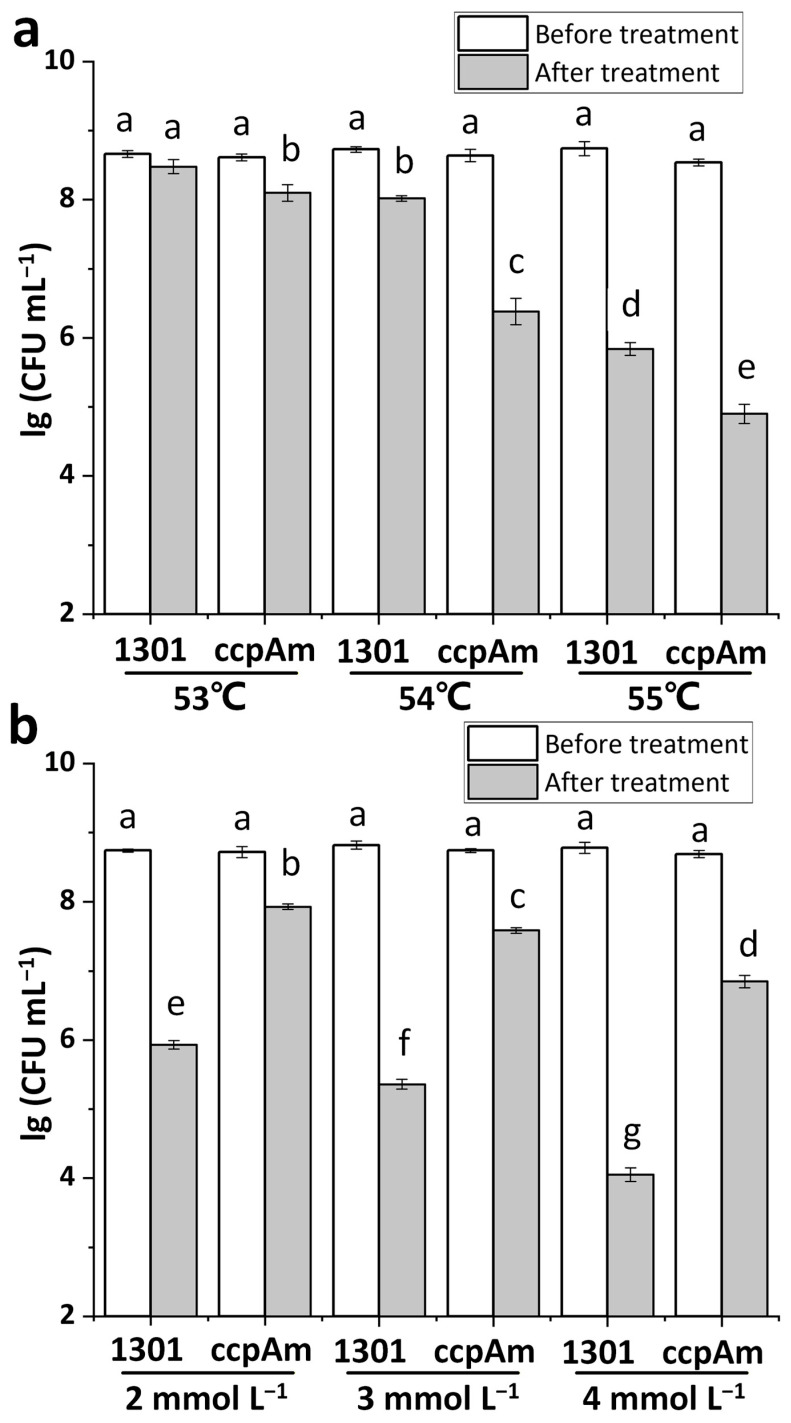
Heat and oxidative stress tolerance of *L. rhamnosus* ΔccpA. (**a**) Heat stress tolerance. (**b**) Oxidative stress tolerance. ^a–g^ Values within each column with different superscripts are significantly different (*p* < 0.05).

When the OD_600_ reached approximately 1.8 (using a biophotometer), the viable cell counts of both *L. rhamnosus* hsryfm 1301 and ΔccpA were in the range of 8.55–8.75 lg (CFU mL^−1^). After treatment at 53 °C, 54 °C, and 55 °C, the viable counts of *L. rhamnosus* hsryfm 1301 decreased to 8.48, 8.02, and 5.84 lg (CFU mL^−1^), respectively. In contrast, ΔccpA exhibited decreased viable counts of 8.10, 6.38, and 4.90 lg (CFU mL^−1^), respectively (Figure 2a). These results demonstrate that the *ccpA* knockout reduced the heat stress tolerance of ΔccpA. Following treatment with 2 mmol L^−1^, 3 mmol L^−1^, and 4 mmol L^−1^ H_2_O_2_, the viable counts of *L. rhamnosus* hsryfm 1301 decreased to 5.93, 5.36, and 4.06 lg (CFU mL^−1^), respectively. Conversely, ΔccpA showed significantly higher tolerance, with viable counts decreasing only to 7.93, 7.59, and 6.85 lg (CFU mL^−1^), respectively (Figure 2b). This indicates that the *ccpA* knockout enhanced the oxidative stress tolerance of ΔccpA across all three H_2_O_2_ concentrations. The *ccpA* gene is thus a key element in *L. rhamnosus* for tolerating both oxidative and heat stress.

### 3.3. The Impact of ccpA Knockout on Gene Transcription in L. rhamnosus

**Figure 3:**
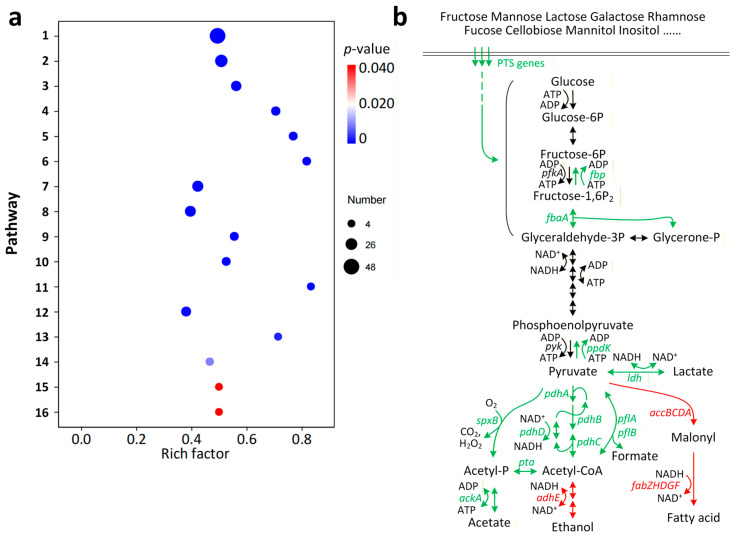
Genes regulated by the deletion of *ccpA* (ΔccpA compared with hsryfm 1301). (**a**) KEGG enrichment analysis of DEGs. Rich factor is the ratio of DEG number enriched in one pathway to the total gene number of this pathway. The size of circle dot means gene number. Pathway: 1, Phosphotransferase system (PTS); 2, Fructose and mannose metabolism; 3, Pyruvate metabolism; 4, Fatty acid biosynthesis; 5, Propanoate metabolism; 6, Inositol phosphate metabolism; 7, Galactose metabolism; 8, Starch and sucrose metabolism; 9, Pentose and glucuronate interconversions; 10, Ascorbate and aldarate metabolism; 11, Staphylococcus aureus infection; 12, Glycolysis/Gluconeogenesis; 13, Cationic antimicrobial peptide (CAMP) resistance; 14, Carbon fixation pathways in prokaryotes; 15, Citrate cycle (TCA cycle); 16, Biotin metabolism. (**b**) The key genes with altered expression in the Pyruvate metabolism and Glycolysis/Gluconeogenesis pathways. Red arrows: downregulation; Green arrows: upregulation; Black arrows: not significantly regulated.

*L. rhamnosus* hsryfm 1301 and ΔccpA were cultured in MRS medium. When the OD_600_ reached approximately 1.8, transcriptomic data were determined using RNA sequencing technology. In ΔccpA, no detectable transcription of the *ccpA* gene was observed, further confirming the successful knockout of *ccpA*. Compared to the wild-type strain, 260 genes were upregulated and 55 genes were downregulated in the *ccpA* deficient strain. However, typical stress tolerance genes, such as the heat shock protein gene (*hsp20*), ATP-dependent protease genes (*clpP*, *clpC*, *clpX*, *clpL*), and molecular chaperone genes (*groEL*, *groES*, *dnaK*), were not included among them. KEGG analysis revealed enrichment in 16 metabolic pathways, including phosphotransferase system (PTS), fructose and mannose metabolism, pyruvate metabolism, fatty acid biosynthesis, propanoate metabolism, inositol phosphate metabolism, galactose metabolism, starch and sucrose metabolism, pentose and glucuronate interconversions, and ascorbate and aldarate metabolism (Figure 3a).

The overwhelming majority (184 out of 199) of the regulated carbon source transport and metabolism genes were upregulated, including 10 genes related to ascorbate and aldarate metabolism. Conversely, among the 12 regulated fatty acid synthesis genes, the *accBCDA* genes responsible for generating malonyl-CoA and the *fabZHacpPfabDGF* genes involved in fatty acid synthesis are downregulated. In the glycolysis/gluconeogenesis pathway, the *pfkA* gene (encoding 6-phosphofructokinase for fructose-1,6-bisphosphate synthesis) and the *pyk* gene (encoding pyruvate kinase for the conversion of phosphoenolpyruvate (PEP) to pyruvate) were not significantly regulated. However, the genes for their reverse reactions, *fbp* (encoding fructose-1,6-bisphosphatase) and *ppdK* (encoding phosphate dikinase), were upregulated. The *ppdK* gene was upregulated 28-fold. In the Pyruvate metabolism pathway, genes related to formate metabolism (*pflA*, *pflB*), acetyl-CoA generation (*pdhDCBA*), acetate generation (*pta*, *ackA*), and pyruvate oxidation (*spxB*) were upregulated. While the *adhE* gene, which consumes NADH, was downregulated (Figure 3b). In other aspects, the rate-limiting enzyme of hexosamine synthesis, *glmS*, was strongly downregulated. The *nagA* gene, which catalyzes the deacetylation of N-acetylglucosamine-6-phosphate (GlcNAc-6-P), and the *glgC* gene, which catalyzes the reaction of ATP and α-D-glucose-1-phosphate to produce ADP-glucose and pyrophosphate, are upregulated by 3.86 and 10.58 times, respectively. Overall, the strongest upregulation observed was approximately 2000-fold, while the strongest downregulation was 7%.

### 3.4. Changes in Heat and Oxidative Stress Tolerance of ΔccpA Under Different Nitrogen Conditions

**Figure 4:**
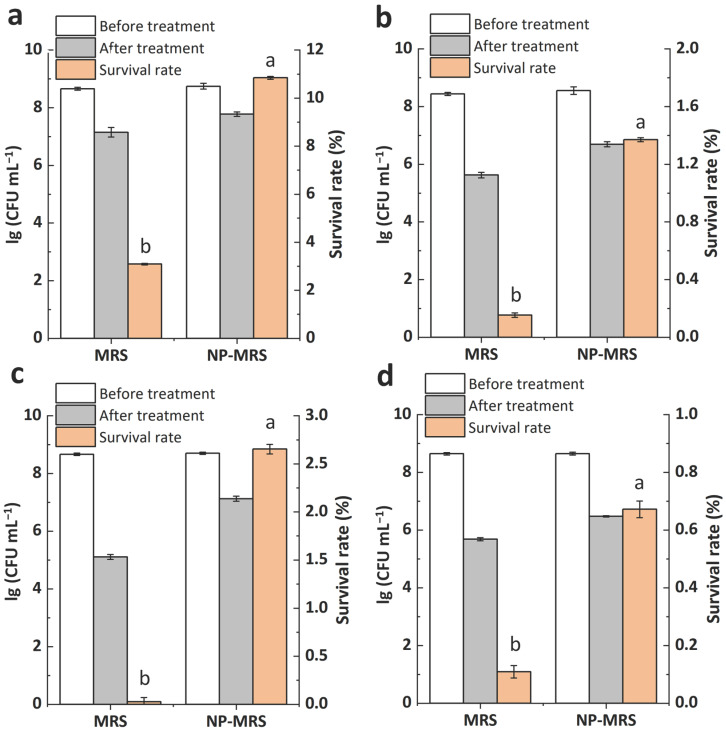
Heat stress tolerance of L. rhamnosus strains in MRS and NP-MRS. (**a**) hsryfm 1301, 55 °C, 1 h. (**b**) ΔccpA, 55 °C, 1 h. (**c**) ∆ccpA/pccpA(L.r.)+, 56 °C, 1 h. (**d**) ∆ccpA/pccpA(L.p.)+, 56 °C, 1 h. ^a^^,^^b^ Values within each column with different superscripts are significantly different (*p* < 0.05).

Given the pronounced effect of *ccpA* on the strain’s heat and oxidative stress tolerance, differential treatment intensities were employed for the various strains to facilitate the successful acquisition of their stress tolerance variances across different nitrogen source conditions. In terms of heat stress tolerance, the wild-type strain exhibited survival rates of 3.09% in MRS and 10.84% in NP-MRS (Figure 4a), indicating that a tryptone-free environment significantly enhanced its heat stress tolerance. Similarly, the *ccpA* deficient strain (ΔccpA) also showed higher heat stress tolerance in NP-MRS compared to standard MRS (Figure 4b). The complemented strains exhibited the same trend in tolerance changes (Figure 4c,d). This indicates that the enhancing effect of a tryptone-free environment on the heat stress tolerance of *L. rhamnosus* was still present in the *ccpA* deficient mutant.

**Figure 5:**
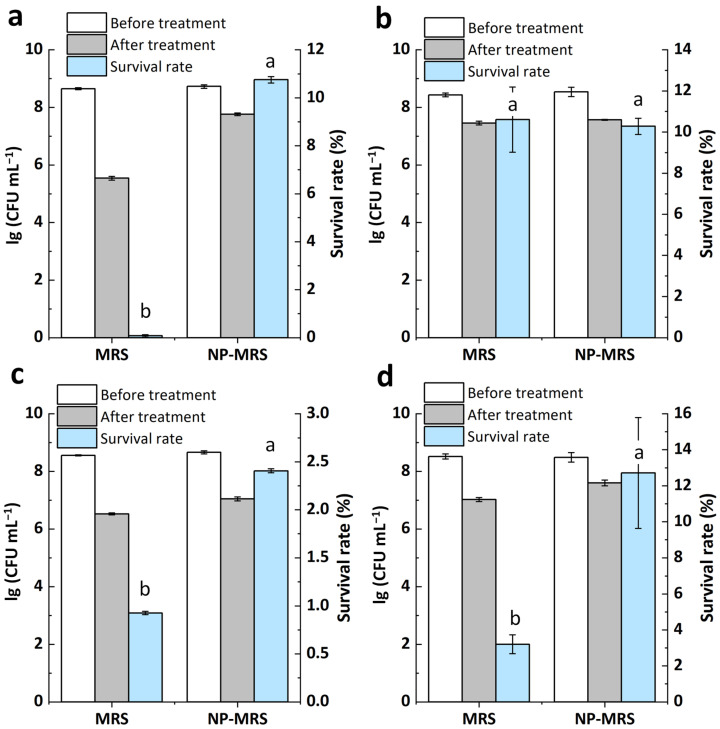
Oxidative stress tolerance of *L. rhamnosus* strains in MRS and NP-MRS. (**a**) hsryfm 1301, 3 mmol L^−1^ H_2_O_2_, 1 h. (**b**) ΔccpA, 4 mmol L^−1^ 1 h. (**c**) ∆ccpA/pccpA(L.r.)+, 3 mmol L^−1^, 1 h. (**d**) ∆ccpA/pccpA(L.p.)+, 4 mmol L^−1^, 1 h. ^a,b^ Values within each column with different superscripts are significantly different (*p* < 0.05).

Regarding oxidative stress tolerance, the wild-type strain exhibited survival rates of 0.07% in MRS and 10.78% in tryptone-free MRS (Figure 5a). The tryptone-free environment significantly enhanced its oxidative stress tolerance. In contrast, ΔccpA showed an oxidative stress survival rate of 10.28% in NP-MRS and 10.79% in MRS (Figure 5b). Its survival rates in these two media were identical, indicating that the effect of nitrogen source on oxidative tolerance was abolished in the mutant. Moreover, in two complemented strains, the phenomenon of low-nitrogen enhancing oxidative stress tolerance was re-established, resulting in a trend consistent with the wild-type strain (Figure 5c,d). Therefore, the tryptone-free environment likely regulates the oxidative stress tolerance of *L. rhamnosus* through CcpA.

### 3.5. Effect of ccpA Gene Knockout on the Nitrogen Source Response in L. rhamnosus

The total number of genes regulated by tryptone was lower in the wild-type strain compared to the mutant strain, with 70 and 101 genes, respectively. The deletion of the *ccpA* gene caused a similar upregulation of carbon utilization and transport pathways in NP-MRS as in MRS. However, the number of regulated genes in these pathways was lower than in MRS (Table 3).

At the pathway level, purine metabolism and pyrimidine metabolism pathways were enriched in both the YM (wild-type in MRS) vs. YN (wild-type in NP-MRS) and QM (∆ccpA in MRS) vs. QN (∆ccpA in NP-MRS) groups, indicating that the transcription of these pathways was affected by nitrogen sources. Under low-nitrogen conditions, purine metabolism pathway was primarily upregulated, while pyrimidine metabolism pathway was primarily downregulated. Notably, in the YN vs. QN group, the purine metabolism pathway was also significantly regulated. In the wild-type strain, the fatty acid biosynthesis pathway was unaffected by the nitrogen source, but it was upregulated in the *ccpA* deficient strain (Table 3). This indicates that both purine metabolism and fatty acid biosynthesis pathways are simultaneously influenced by the *ccpA* gene and the nitrogen source environment. In the YM vs. YN group, 12 PTS sugar transport-related genes were upregulated but not enriched.

**Figure 6:**
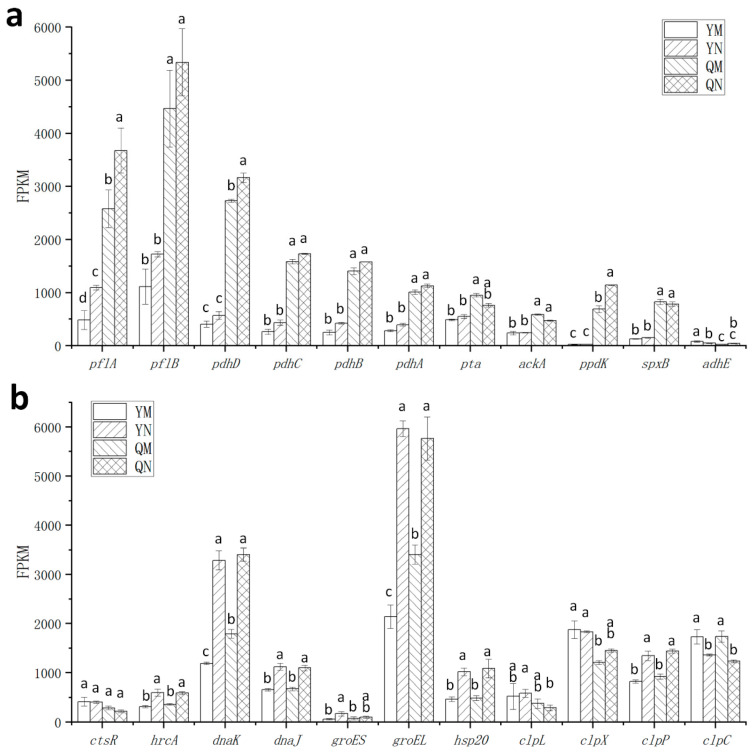
Transcriptional levels of key genes related to acetate production and typical stress tolerance. (**a**) Genes related to acetate production. (**b**) Typical stress tolerance genes. ^a–d^ Values within each column with different superscripts are significantly different (*p* < 0.05). YM, wild-type in MRS; YN, wild-type in NP-MRS; QM, ∆ccpA in MRS; QN, ∆ccpA in NP-MRS.

**Figure 7:**
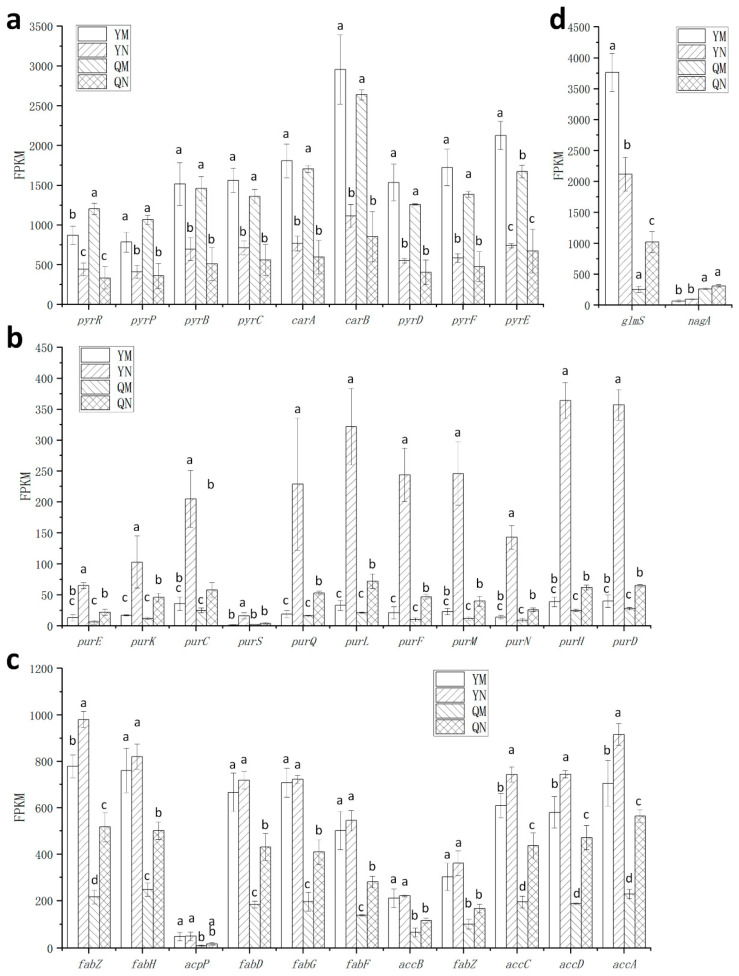
Transcriptional levels of key genes potentially associated with heat and oxidative stress tolerance. (**a**) Genes in the de novo pyrimidine synthesis operon (*pyr* operon). (**b**) Genes in the de novo purine synthesis operon (*pur* operon). (**c**) Genes in the fatty acid synthesis operon (*fab* operon). (**d**) Genes related to hexosamine pathway. ^a–d^ Values within each column with different superscripts are significantly different (*p* < 0.05). YM, wild-type in MRS; YN, wild-type in NP-MRS; QM, ∆ccpA in MRS. QN; ∆ccpA in NP-MRS.

From an expression perspective, the genes involved in pyruvate metabolism were largely unaffected by the nitrogen source, but were upregulated in the mutant strain across different nitrogen conditions (Figure 6a). For typical stress tolerance genes, the transcription of *groEL* and *dnaK* was significantly upregulated by the tryptone-free environment. Other genes, such as *hrcA*, *dnaJ*, *clpP*, and *hsp20*, were also influenced by the tryptone-free environment (though their fold changes did not reach 2-fold), and this regulation was unaffected by the absence of the *ccpA* gene (Figure 6b). The pyrimidine de novo biosynthesis operon genes were downregulated by tryptone, with the same trend in both the wild-type and mutant strains (Figure 7a). Conversely, the genes of the purine de novo synthesis operon were upregulated by tryptone in both strains. However, the regulatory effect of the tryptone-free environment on the purine de novo synthesis operon genes was significantly stronger in the wild-type strain than in the mutant strain (Figure 7b), indicating that *ccpA* enhanced nitrogen source-mediated regulation of purine de novo biosynthesis. The tryptone-induced downregulation of fatty acid biosynthesis genes occurred only in MRS broth and not in low-nitrogen conditions (Figure 7c). Notably, the *glms* gene was downregulated by tryptone in the wild-type strain but upregulated in the mutant strain (Figure 7d). These findings demonstrate that *ccpA* exerts gene-specific effects on the nitrogen source response.

### 3.6. Effects of Cytosine on the Heat and Oxidative Stress Tolerance of L. rhamnosus

**Figure 8:**
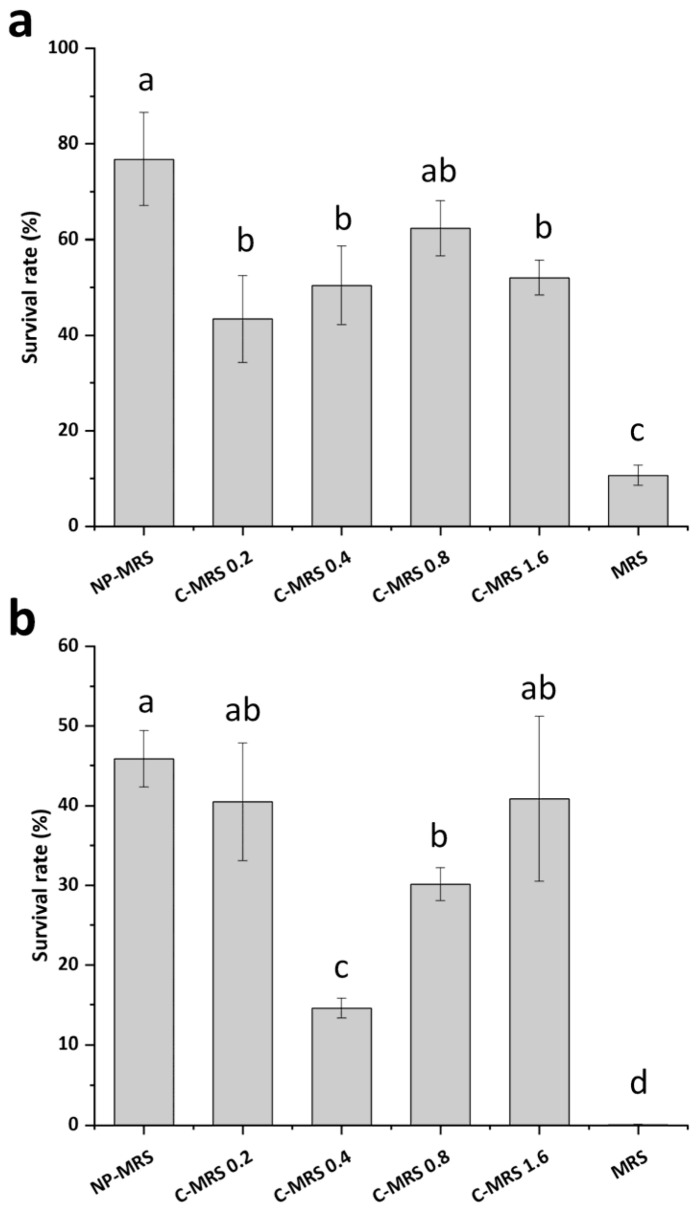
Heat and oxidative stress tolerance of *L. rhamnosus* hsryfm 1301 in NP-MRS supplemented with cytosine. (**a**) Heat stress, 54 °C, 1 h. (**b**) Oxidative stress, 3 mmol L^−1^ H_2_O_2_, 1 h. C-MRS 0.2, NP-MRS supplemented with 0.2 g L^−1^ cytosine; C-MRS 0.4, NP-MRS supplemented with 0.4 g L^−1^ cytosine; C-MRS 0.8, NP-MRS supplemented with 0.8 g L^−1^ cytosine; C-MRS 1.6, NP-MRS supplemented with 1.6 g L^−1^ cytosine. ^a–d^ Values within each column with different superscripts are significantly different (*p* < 0.05).

To investigate the impact of nucleotide metabolism on the heat and oxidative stress tolerance of *L. rhamnosus*, varying concentrations of cytosine were added to NP-MRS. The results showed that the addition of cytosine significantly reduced the heat stress tolerance of *L. rhamnosus*. Notably, when the cytosine concentration exceeded 0.2 g L^−1^, the heat stress tolerance of *L. rhamnosus* began to recover with increasing cytosine concentrations (Figure 8a). At 0.4 g L^−1^, cytosine reduced the oxidative stress survival rate of *L. rhamnosus* to approximately 15%. Higher concentrations of cytosine had a diminished effect on the oxidative stress tolerance of *L. rhamnosus* (Figure 8b). Importantly, in the cytosine-supplemented broth, *L. rhamnosus* showed significantly higher tolerance to heat and oxidative stress than in MRS broth.

## 4. Discussion

The carbon catabolite repression (CCR) in homolactic fermentation LAB (such as *L. plantarum*, *L. delbrueckii*, *Lc. lactis*, etc.) is dependent on the *ccpA* gene [15,29,30]. The *ccpA* gene regulates crucial physiological activities in these LAB, including nutrient utilization, growth, and stress tolerance, which are highly relevant to food production and development. However, the *ccpA* gene in *L. rhamnosus*, which is one of the most widely used probiotics, has not been studied.

In this study, an unmarked *ccpA* mutant of *L. rhamnosus* hsryfm 1301, ΔccpA, was constructed. After *ccpA* deletion, the mutant exhibited slow growth and a 10-h-delayed stationary phase, but the final biomass was not significantly affected. Complementation with the *ccpA* gene from either *L. rhamnosus* or *L. paracasei* restored the growth rate to the wild-type level. This suggests that, similar to *Lc. lactis*, *L. plantarum*, and *L. delbrueckii* [15,29,30], the *ccpA* gene increases the growth rate of *L. rhamnosus*, which may help it rapidly occupy ecological niches. The *ccpA* genes from *L. rhamnosus* and *L. paracasei* could play a similar role.

In *L. rhamnosus*, the deletion of *ccpA* led to the upregulation of 46 genes related to carbon source transport or involved in the utilization of fructose, mannose, galactose, starch, sucrose, and pentoses. This highlights the *ccpA* gene’s key role in CCR of *L. rhamnosus*. In *Lc. lactis*, several genes related to glycolysis (*pfk*, *pyk*, *ldh*) are upregulated by CcpA [17]. Similarly, in *L. plantarum* and *L. delbrueckii*, the same four glycolysis and lactate production genes (*pfk*, *pyk*, *pgk*, *ldh*) are significantly downregulated in *ccpA* mutants [15,16]. This downregulation is likely the main reason for the slower growth of the *ccpA* mutants in these three LAB species. However, in *L. rhamnosus* ΔccpA, *pfk*, *pyk*, and *pgk* were not downregulated, and *ldh* was even upregulated. Instead, the gluconeogenesis genes *fbp* (reverse reaction of *pfk*) and *ppdK* (reverse reaction of *pyk*) were upregulated. Therefore, in *L. rhamnosus*, CcpA enhances growth rate not by upregulating glycolysis, but by repressing gluconeogenesis. Additionally, GlmS is the rate-limiting enzyme in the hexosamine pathway, providing precursor molecules for the biosynthesis of peptidoglycan, essential components of bacterial cell walls [31]. The *glmS* gene was strongly downregulated in *L. rhamnosus* ΔccpA, with transcript levels only 7% of the wild-type strain, and the transcription of the fatty acid synthesis operon was also downregulated, which may also contribute to the reduced growth rate.

The regulatory scope of CcpA is broad; thus, its deletion impacts not only nutrient utilization and cell growth but also stress tolerance and environmental adaptation [15,16,17,18]. *L. rhamnosus* ΔccpA showed significantly stronger tolerance to oxidative stress at various intensities, but lower survival rates under heat stress conditions of varying intensities. Similarly, the *ccpA* deficient strain of *L. plantarum* exhibited stronger oxidative stress tolerance but lower tolerance to osmotic, cold, heat, and starvation stresses than the wild-type strain [19,32]. The *L. delbrueckii ccpA* mutant exhibited the same trend of changes in heat and oxidative stress tolerance [29]. For applications where oxidative stress is the primary concern, selecting natural variants or constructing strains with attenuated CcpA activity could be beneficial. Conversely, for processes involving heat stress, strains with fully functional CcpA are preferable. This highlights the potential for stress-specific starter culture selection.

In the *ccpA* deficient strain of *L. plantarum*, the decrease in heat stress tolerance was accompanied by decreased transcription of class I heat shock response genes, particularly *dnaK*, *groEL*, *grpE*, *clpL*, and *clpE*, which was confirmed by proteomic data [19,33,34]. These genes are related to oxidative stress and heat stress tolerance and may response to freeze-drying [35]. Furthermore, in the *L. delbrueckii ccpA* mutant, *hrcA* was upregulated, while *tuf*, *dnaK*, and *groEL* were downregulated [16]. These data point to the hypothesis that the reduced heat stress tolerance in *ccpA* mutants is related to the regulation of class I heat shock response genes. However, in *L. rhamnosus* ΔccpA, genes such as *hrcA*, *groEL*, *groES*, *dnaK*, *hsp20*, *clpP*, *clpC*, *clpX*, *clpL*, *tuf*, and *ctsR* were not regulated. This suggests that the reduced heat stress tolerance of *L. rhamnosus* was not caused by these typical stress tolerance genes, but rather by the transcriptional regulation of other genes.

Genes related to ROS detoxification, such as catalase and superoxide dismutase, were not found in the *L. rhamnosus* genome [36]. The deletion of *ccpA* also did not cause transcriptional changes in DNA repair genes (e.g., *recA*, *uvrA*, *uvrB*). In *L. rhamnosus*, pyruvate metabolism genes are upregulated under oxidative conditions [37]. Pyruvate metabolism genes were extensively regulated in ΔccpA. Genes in multiple pathways related to acetate production (*phdDCBA*, *spxB/pox*, *pflAB*, *ackA*) were upregulated. In *L. plantarum*, the regulation of pox and ack in mixed acid metabolism was consistent under aerobic cultivation, non-preferred carbon source cultivation, and *ccpA* deletion, along with *nox* (NADH oxidase gene) and *npr* (NADH peroxidase gene) involved in NAD/NADH cycling [15,19,38,39]. These pathways compete with LDH and can reduce NADH consumption. Furthermore, the NADH-consuming *adhE* and fatty acid synthesis pathways were downregulated in *L. rhamnosus* ΔccpA. Correspondingly, one of the two NADH oxidase genes was upregulated. Therefore, the enhanced oxidative stress tolerance in the *L. rhamnosus ccpA* mutant might be related to NADH/NAD balance. While the relative ratios of NADH/NAD were not directly measured in this study, it is a compelling hypothesis that the observed enhanced oxidative stress tolerance is facilitated by these metabolic adjustments. Future studies quantifying these metabolite pools will be critical to validate this proposed mechanism and to fully elucidate how CcpA-mediated regulation influences the redox economy of *L. rhamnosus*. Additionally, SpxB/Pox generates H_2_O_2_ when utilizing pyruvate, which could potentially pre-adapt the *ccpA* mutant to oxidative stress, thereby enhancing its tolerance.

LAB often live in nutrition-rich environments, relying mainly on external oligopeptide uptake for nitrogen sources and having weak amino acid self-synthesis ability [40]. Amino acids play a positive role in the tolerance of LAB to stresses such as acid, oxidation, and osmotic pressure. For instance, the decarboxylation of glutamate and aspartate, as well as the deamination of arginine, serve as crucial pathways for LAB to resist acid stress [41]. To combat oxidative stress, the reduction in cysteine is helpful [7]. Previous studies showed that low-nitrogen cultivation improves the heat and oxidative stress tolerance of *L. rhamnosus* [12,22]. GlnR is the global regulatory element for nitrogen source metabolism in lactobacilli. In environments with high amino acid or AMP concentrations, GlnR, assisted by GlnA, suppresses the transcription of genes related to nitrogen source transport, peptidases, and amino acid metabolism [42]. GlnR in Levilactobacillus brevis is associated with acid stress tolerance [43]. However, *glnR* in *L. rhamnosus* hsryfm 1301 only affects induced heat and oxidative stress tolerance (e.g., via sublethal preadaptation), but has no impact on direct heat/oxidative shock resistance [13].

After *ccpA* knockout, the *ccpA* mutant no longer showed enhanced oxidative stress tolerance under low nitrogen conditions. Complementation with the *ccpA* gene from either *L. rhamnosus* or *L. paracasei* restored the enhancing effect of low nitrogen cultivation on oxidative stress tolerance. In contrast, *L. rhamnosus* ΔccpA still exhibited higher heat stress tolerance under low nitrogen conditions. This suggests that the effect of nitrogen source on oxidative stress tolerance is mediated through the *ccpA* gene. This study reconfirmed that low-nitrogen conditions significantly enhance the heat stress and oxidative stress tolerance of *L. rhamnosus*. Modulating the nitrogen composition, specifically by reducing the concentration of tryptone or certain amino acids in the fermentation medium could be a viable approach to improve bacterial survival during subsequent processing stresses like spray drying, heat treatment, and storage. However, this enhancement effect depends on the *ccpA* gene. Therefore, the optimization of carbon sources should not be overlooked when adjusting the nitrogen source composition of the culture medium.

In both MRS and NP-MRS, *L. rhamnosus* hsryfm 1301 and ΔccpA showed strong consistency in the regulation of carbon source transport and utilization pathways, although the number of regulated genes within these pathways decreased. This indicates that the *ccpA* gene performs its CCR function under both conditions. The expression of genes related to mixed-acid metabolism (*phdDCBA*, *spxB/pox*, *pflAB*, *ackA*) was also unaffected by the nitrogen source environment. However, under low nitrogen conditions, the regulation of some genes was relieved. The downregulating effect of *ccpA* deletion on the fatty acid synthesis operon and *glmS* gene was weakened by low nitrogen conditions. These genes in the *ccpA* mutant were upregulated under low nitrogen. This indicates they are co-regulated by CcpA and nitrogen availability. The fatty acid synthesis operon directly affects the fatty acid composition of the cell membrane, thereby influencing membrane fluidity and stability [44], which may relate to heat stress tolerance. Furthermore, *dnaK* and *groEL* in *L. rhamnosus* hsryfm 1301 were both upregulated by low nitrogen conditions, and this upregulation was unaffected by the *ccpA* gene deletion, potentially also linked to changes in heat stress tolerance. The fluctuations in heat stress tolerance caused by *ccpA* deletion and changes in nitrogen source environment may result from distinct gene expression changes, whose specific functions require further study.

Various amino acid supplementations reverse the low-tryptone phenotypes of *L. rhamnosus* [12]. Nucleic acid metabolism is a downstream pathway of amino acid metabolism. A particularly intriguing finding was the differential regulation of the de novo purine and pyrimidine biosynthesis pathways by the interplay between CcpA and nitrogen availability. The de novo synthesis pathways of purine and pyrimidine in both *L. rhamnosus* hsryfm 1301 and ΔccpA were significantly regulated by nitrogen source, but in opposite directions. The de novo purine synthesis was upregulated under low nitrogen, while the de novo pyrimidine synthesis was downregulated. This suggests that the role of CcpA and nitrogen sensing extends beyond biomass production. Notably, transcript levels revealed that the upregulating effect of low nitrogen on de novo purine synthesis was significantly greater in the wild-type than in the *ccpA* mutant, indicating that the de novo purine synthesis pathway is co-regulated by CcpA and nitrogen availability, and *ccpA* deletion weakens nitrogen source regulation of the *pur* operon. We propose that the energy-intensive nature of purine synthesis makes it more dependent on the efficient carbon catabolism enforced by CcpA, explaining its upregulation when carbon flux is optimal. In *L. plantarum*, inactivation of *ccpA* also affects nucleotide metabolism, with its *pyr* operon being downregulated. Researchers speculated that CcpA might regulate the pyrimidine synthesis pathway by upregulating CO_2_ concentration or binding directly to the regulatory region [34]. After heat or oxidative stress pretreatment, *L. rhamnosus* hsryfm 1301 downregulates cytosine uptake genes [13]. Since de novo pyrimidine and purine synthesis pathways share common substrates, PRPP and glutamine, it is hypothesized that the upregulation of the *pur* operon in *L. rhamnosus* might have a similar effect to the downregulation of the *pyr* operon, namely increasing purine concentration and decreasing pyrimidine concentration. Adding 0.4–0.8 g L^−1^ cytosine to NP-MRS reduced both the heat and oxidative stress tolerance of *L. rhamnosus* hsryfm 1301, but the reduction was less than in medium supplemented with tryptone. This suggests that changes in nucleotide concentration might be one reason for amino acid-induced changes in stress tolerance, but other mechanisms exist. Nevertheless, this is a novel finding that the heat and oxidative stress tolerance of *L. rhamnosus* in foods might be enhanced by avoiding the use of components with high pyrimidine content. The reasons why oxidative stress tolerance in the *L. rhamnosus ccpA* mutant is unaffected by nitrogen concentration may include: (1) the deletion of *ccpA* relieved or weakened gene regulation; (2) the upregulation of mixed-acid metabolism genes caused by *ccpA* deletion masked the effect of nitrogen-regulated genes.

## 5. Conclusions

This study investigated the heat and oxidative stress tolerance, along with transcriptome changes, of *L. rhamnosus* and its *ccpA* mutant under different nitrogen source conditions. The *ccpA* gene is the central regulator of CCR in *L. rhamnosus*. Its presence enhances the growth rate of *L. rhamnosus*, maintains its homolactic fermentation pattern, improves its heat stress tolerance, and reduces its oxidative stress tolerance. Fatty acid metabolism and purine metabolism in *L. rhamnosus* are co-regulated by the *ccpA* gene and nitrogen levels. After *ccpA* deletion, the nitrogen source-induced changes in oxidative stress tolerance disappeared. The heat and oxidative stress tolerance of *L. rhamnosus* is determined by a complex metabolic network. This study further investigates the stress tolerance mechanisms of *L. rhamnosus* from a metabolic regulation perspective. The viability of LAB during food processing, such as heat treatment, dehydration, and spray drying, can be maintained by controlling the concentration of tryptone or the types of sugars.

## Figures and Tables

**Table 2:** Primers used in this study.

Primer	Sequence (5′-3′)	Restriction Site
Eco-ccpaupF	TTTGAATTCCATATACCCATGATTGTCGGTGC	*Eco*R I
ccpaupR	TTTATTTTCTCCTTAGTCGTGAAAA	
ccpadownF	TTTTCACGACTAAGGAGAAAATAAAACAGAAGTAACACGATATTCTGGC	
hind-ccpadownR	AAGAAGCTTGTCATCAAGTCAAAAAGACCAAG	*Hind* III
ccpaTestF	GTCCATCGCGGTTAAGTTAGCC	
ccpaTestR	CAACATTCGCCAATCAAGGTG	
M13c-F	CCCAGTCACGACGTTGTAAAACG	
RV-M	GAGCGGATAACAATTTCACACAGG	
Eco-1301ccpAF	GGAATTCGGCTACTCCTTAAAACTCGCTG	*Eco*R I
Hind-1301ccpAR	ATTAAGCTTCTACTTGGTTGAACCACGCTTC	*Hind* III
pMG36eF	TAATTCGAGCTCGCCCGG	
pMG36eR	ACCGAATTCGATCGACCCATA	
36e-FGL-ccpAF	TATGGGTCGATCGAATTCGGTCAATCAAGCATCGTGGTAAAATAG	
36e-FGL-ccpAR	CCGGGCGAGCTCGAATTATTATTTCGTTGAACCACGCTTC	

**Table 3:** Distribution of upregulated and downregulated genes in the four pair-wise comparisons based on KEGG pathway enrichment.

YM vs. QM			YN vs. QN			YM vs. YN			QM vs. QN		
Pathways	U *	D *	Pathways	U *	D *	Pathways	U *	D *	Pathways	U *	D *
Phosphotransferase system (PTS)	46	2	Phosphotransferase system (PTS)	33	2	Purine metabolism	13	1	Fatty acid biosynthesis	9	0
Fructose and mannose metabolism	28	2	Fructose and mannose metabolism	20	2	Pyrimidine metabolism	2	9	Pyrimidine metabolism	3	9
Pyruvate metabolism	12	6	Inositol phosphate metabolism	8	0	beta-Lactam resistance	5	0	Purine metabolism	13	0
Fatty acid biosynthesis	0	12	Ascorbate and aldarate metabolism	10	0	Alanine, aspartate and glutamate metabolism	1	4	beta-Lactam resistance	5	1
Propanoate metabolism	6	4	Staphylococcus aureus infection	5	0	Tuberculosis	2	0	Quorum sensing	9	0
Inositol phosphate metabolism	9	0	Galactose metabolism	17	0	RNA degradation	2	1	Alanine, aspartate and glutamate metabolism	2	3
Galactose metabolism	22	0	Starch and sucrose metabolism	17	0	Quorum sensing	5	0	Biotin metabolism	3	0
Starch and sucrose metabolism	21	0	Cationic antimicrobial peptide (CAMP) resistance	5	0				ABC transporters	10	1
Pentose and glucuronate interconversions	10	0	Pyruvate metabolism	11	0				Prodigiosin biosynthesis	2	0
Ascorbate and aldarate metabolism	10	0	Glycolysis/Gluconeogenesis	13	0				Propanoate metabolism	3	0
Staphylococcus aureus infection	5	0	Purine metabolism	0	13						
Glycolysis/Gluconeogenesis	15	1	Pentose and glucuronate interconversions	7	0						
Cationic antimicrobial peptide (CAMP) resistance	5	0	Citrate cycle (TCA cycle)	4	0						
Carbon fixation pathways in prokaryotes	3	4	Propanoate metabolism	5	0						
Citrate cycle (TCA cycle)	4	0									
Biotin metabolism	0	4									

* U: Number of genes upregulated; D: Number of genes downregulated; YM, wild-type in MRS; YN, wild-type in NP-MRS; QM, ∆ccpA in MRS; QN, ∆ccpA in NP-MRS.

## Data Availability

The raw sequence data have been deposited in the Genome Sequence Archive at National Genomics Data Center (GSA: CRA027319, CRX1820520-CRX1820531; publicly accessible at https://ngdc.cncb.ac.cn/gsa, accessed on 9 October 2025).

## Abbreviations

The following abbreviations are used in this manuscript:

LAB	Lactic acid bacteria
KEGG	Kyoto Encyclopedia of Genes and Genomes
DEGs	differentially expressed genes
FPKM	fragments per kilobase of exon model per million mapped fragments
PTS	Phosphotransferase system
CCR	carbon catabolite repression
ROS	reactive oxygen species
